# Atypical Presentation of Homozygous UROD Mutation: Porphyria Cutanea Tarda or Mild Hepatoerythropoietic Porphyria?

**DOI:** 10.1111/cge.70007

**Published:** 2025-06-19

**Authors:** Pedro Gabriel Dotto, Mônica Ribeiro de Azevedo Vasconccellos, José Francisco da Silva Franco, Caio Perez Gomes, João Bosco Pesquero

**Affiliations:** ^1^ Department of Biophysics Federal University of São Paulo São Paulo Brazil; ^2^ Department of Dermatology Federal University of São Paulo São Paulo Brazil

**Keywords:** genetic testing, hepatoerythropoietic porphyria, porphyria cutanea tarda, uroporphyrinogen decarboxylase

## Abstract

We report a patient homozygous for the *UROD* c.185C>T (p.P62L) variant who presents with clinical features resembling familial porphyria cutanea tarda (PCT). This case highlights the limitations of rigid *UROD*‐related porphyria classifications and supports the existence of a phenotypic continuum modulated by genetic, epigenetic, and environmental factors.

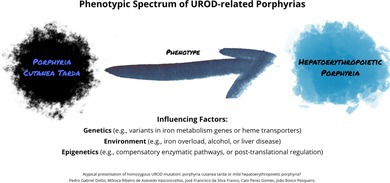

The *UROD* gene (1p34.1) encodes uroporphyrinogen decarboxylase, a key enzyme in the heme biosynthesis. Heterozygous pathogenic *UROD* variants are typically associated with porphyria cutanea tarda (PCT), while homozygous or compound heterozygous variants cause hepatoerythropoietic porphyria (HEP), a rarer and more severe phenotype. Both conditions manifest as non‐inflammatory vesiculobullous lesions, differing in severity, age of onset, and laboratory findings [1]. UROD inhibition is multifactorial, with symptoms emerging when residual activity falls below 20%, as UROD enzymatic activity exceeds that of upstream enzymes [2].

PCT is the most common porphyria and typically appears between the fourth and fifth decades [1]. It is classified into two clinically indistinguishable forms: sporadic and familial. The sporadic form accounts for 70%–80% of cases and is primarily linked to acquired reductions in hepatic UROD activity, often associated with hepatitis C, HIV infection, and alcoholism. The familial form follows an autosomal dominant pattern with low penetrance and decreased UROD activity in all tissues [2]. Standard treatment includes photoprotection, and either phlebotomy or 4‐aminoquinolines. HEP is a rare disorder that usually presents in early childhood with dark urine, photomutilating lesions, dental discoloration, splenomegaly, and hemolytic anemia. Conventional PCT treatments are often ineffective for HEP [1].

We describe a patient homozygous for the *UROD* c.185C>T (p.P62L) variant who presented with features resembling familial PCT. He is a 33‐year‐old Brazilian male, born to reportedly unrelated parents. Symptoms began in childhood with delayed healing of skin lesions, progressing to vesiculobullous eruptions, but without neuropsychomotor impairment. Physical examination is illustrated in Figure 1. Laboratory results showed normal blood count, liver function, and iron metabolism. Tests for hepatitis B, C, and HIV were negative. Notable findings included elevated free erythrocyte protoporphyrin (114 μg/dL, RV ≤ 55 μg/dL) and a urinary porphyrin profile consistent with porphyria, showing marked increases in uroporphyrin (324 μg/24 h, RV ≤ 35), heptacarboxylporphyrin (375 μg/24 h, RV ≤ 20), hexacarboxylporphyrin (25 μg/24 h, RV ≤ 4), and pentacarboxylporphyrin (55 μg/24 h, RV ≤ 2). Coproporphyrins I and III remained within normal limits (36 μg/24 h and 21 μg/24 h, RVs ≤ 73 and ≤ 200, respectively).

**FIGURE 1:**
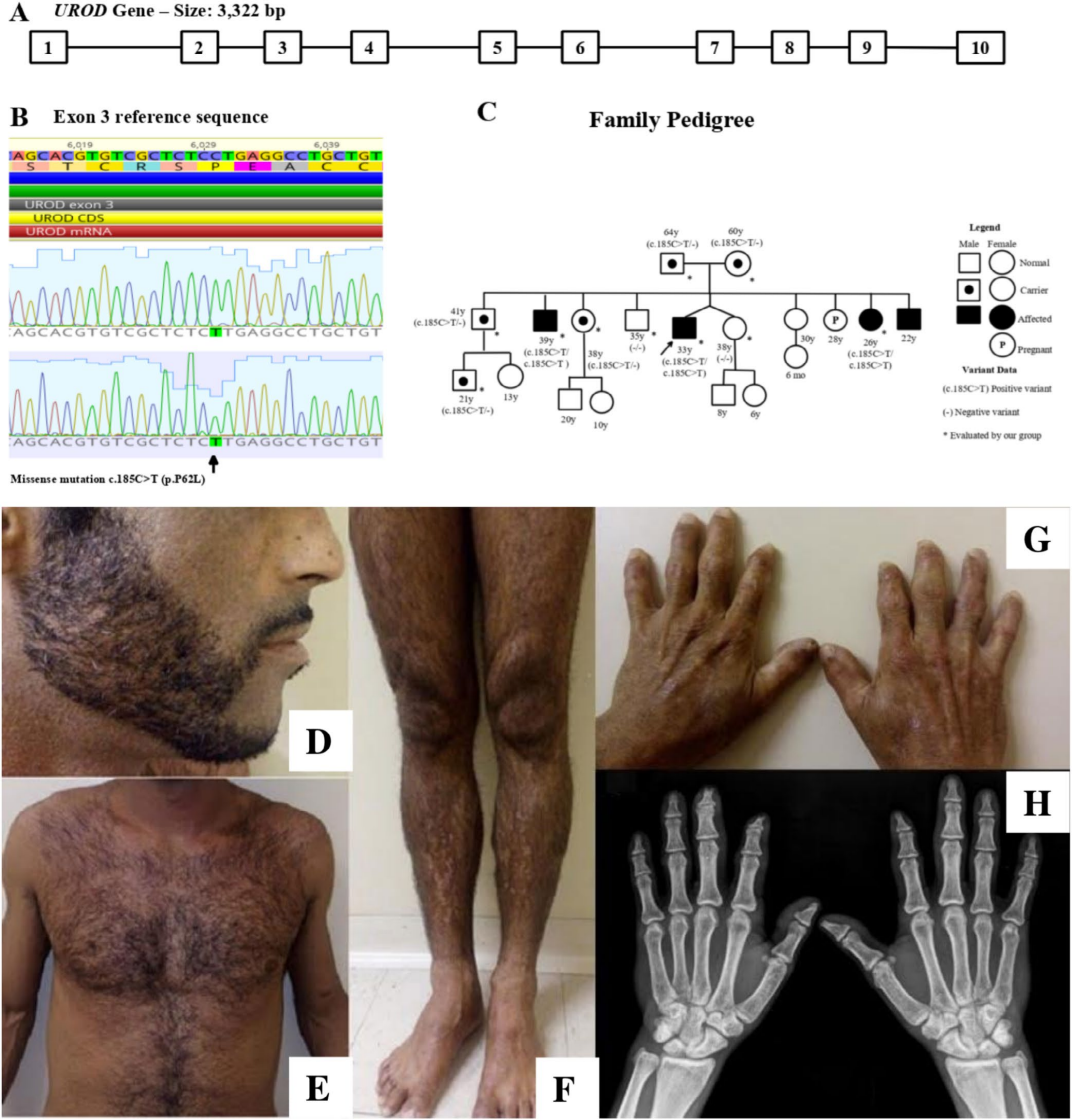
(A) Schematic representation of the *UROD* gene structure. (B) Electropherogram showing the c.185C>T (p.P62L) mutation in affected family members compared to the wild‐type sequence. (C) Family pedigree depicting three generations with affected (black symbols) and unaffected (white symbols) individuals. (D–H) The patient presented with facial skin retraction, hyperpigmentation, hypertrichosis, hand lesions with milia and scars, finger deformities, nail dystrophy, and distal phalangeal bone erosions with joint flexion deformities.

Based on clinical and biochemical findings, the diagnosis was familial PCT. The patient was advised on photoprotection, vaccination, and alcohol restriction. Azelaic acid gel was prescribed for facial hyperpigmentation. The patient and family were referred for genetic counseling and testing. After informed consent, DNA was extracted from peripheral blood. The *UROD* gene was amplified via PCR and analyzed by capillary electrophoresis. Alignment to the reference sequence (GenBank NG_007122) confirmed the pathogenic variant c.185C>T (p.P62L) in homozygosity (Figure 1).

This variant was previously reported in homozygosity only in a Portuguese family with severe, mutilating photosensitivity and marked biochemical abnormalities, consistent with HEP [3]. However, our patient showed a milder phenotype, later onset, no systemic involvement, less skin damage, and a favorable course. Siblings carrying the homozygous variant exhibited similar mild photosensitivity, whereas heterozygous individuals were asymptomatic. A mild HEP phenotype has also been previously reported in a patient with a different homozygous *UROD* mutation [4]. These findings challenge the binary classification of *UROD*‐associated porphyrias, suggesting a phenotypic continuum influenced by genetic, epigenetic, or environmental modifiers.

UROD functions as a homodimer with a (β/α)_8_‐barrel structure, containing an active site cleft per monomer. The c.185C>T (p.P62L) variant affects a proline critical for maintaining structural stability near the catalytic cleft [5]. In vitro studies have shown this mutant has significantly reduced enzymatic activity [3]. However, the clinical variability seen in patients with the same homozygous mutation suggests residual enzyme activity alone does not fully explain disease severity.

Several hypotheses may explain this variability. Genetic modifiers such as variants in iron metabolism genes or heme transporters could alter porphyrin handling [2]. Environmental factors like iron overload, alcohol, or liver disease may also influence expression. Finally, compensatory enzymatic pathways or posttranslational regulation may modulate the mutation's impact.

This case broadens the phenotypic spectrum of the p.P62L variant and raises questions about strict porphyria classification. It highlights the need for further studies assessing *UROD* mutations across genetic and environmental contexts, especially in atypical or overlapping cases.

## Conflicts of Interest

The authors declare no conflicts of interest.

## Peer Review

The peer review history for this article is available at https://www.webofscience.com/api/gateway/wos/peer‐review/10.1111/cge.70007.

## Data Availability

All pertinent data can be found in the centers where the author, J.B.P., is coordinator, and can be made available upon request when duly justified.
